# The Global Pandemic of Falsified Medicines: Laboratory and Field Innovations and Policy Implications

**DOI:** 10.4269/ajtmh.15-0046

**Published:** 2015-06-03

**Authors:** Margaret Hamburg

**Affiliations:** Former Commissioner, U.S. Food and Drug Administration, Silver Spring, Maryland

It is a privilege to introduce this collection of articles in the *American Journal of Tropical Medicine and Hygiene* on the global threat of falsified medicines. Substandard and falsified medical products pose significant risks to global health with potentially devastating and far-reaching consequences. The articles contained in this issue address this threat by exploring new technology for identifying falsified medicines, field innovations for defining their prevalence, and the broader policy implications that demand our attention. It is my hope that this collection contributes to the goal of addressing falsified medicines by identifying not only the nature and scope of a pressing global health problem but also ways to solve it.

Safe, effective, high-quality, and affordable medical products are essential to positive, equitable health outcomes. They are required to prevent, diagnose, and treat our most serious public health threats, and they are necessary for the achievement of the global health goals toward which we have been diligently working for many years—such as accelerated progress toward the Millennium Development Goals and implementation of Universal Health Coverage as envisioned for the post-2015 development agenda.

Globalization, however, has redefined the field of medical product regulation by adding layers of complexity to the supply chain and creating opportunities for the potential contamination and/or intentional adulteration of the ingredients and finished products that pass through its links. Today's medical-product landscape blurs the line between domestic and foreign production, drawing attention to the need for global quality and safety oversight to prevent patient exposure to falsified products.

Regulators have risen to this challenge by pursuing global, evidence-driven strategies and a number of diverse initiatives in the thematic areas addressed by this issue of *AJTMH*. In the area of technology, for example, Food and Drug Administration (FDA) has worked to develop the battery-operated, hand-held Counterfeit Detection Device, which uses ultraviolet light to identify counterfeit drugs.

With respect to understanding the scope and reach of substandard and falsified medicines, the World Health Organization (WHO) has developed a monitoring and surveillance system dedicated to improving global intelligence and data collection to assess the scope, scale, and harm caused by such products. It can provide real-time information on safety trends, enabling the evidenced-based decision making needed to respond quickly and effectively to time-sensitive health risks. Concerted efforts must be made, however, to ensure that this is a robust and sustainable system.

In the policy arena, regulators have also advocated for increased awareness of the problem of substandard and falsified medicines through their work with multilateral organizations. Last year, Member States at the World Health Assembly adopted a resolution aimed at strengthening regulatory systems for medical products, which are fundamental in providing the oversight and enforcement needed to improve supply chain integrity. This resolution is a global health milestone, providing a framework for collective action and representing a comprehensive systems approach that draws attention to emerging priorities and the core components required for effective medicines regulation—including a strong legal foundation, the use of data and technology, human resources, international cooperation, leadership, governance, and sustainable financing.

In all of these endeavors, the development of strong and sustainable partnerships is critical in fostering a targeted and coordinated response to the global threat of falsified medicines. Partnerships are also essential for enhancing regulatory intelligence as well as the coordination and enforcement of regulatory standards. For example, initiatives such as WHO's Member State Mechanism to combat substandard, falsified, and counterfeit products and PAHO's National Regulatory Authorities of Regional Reference provide opportunities to leverage global, regional, and national efforts. And while in its development phase, the International Coalition of Medical Regulatory Authorities is providing leadership, advocacy, and strategic guidance for a range of issues common to medical product regulators worldwide.

As we begin 2015, medical product safety faces new and continuing challenges. Although much hard work remains, this collection of articles underscores the insight, ingenuity, and perseverance of the scholars and practitioners dedicated to making the improvements and changes needed to achieve our collective global health goals and provides critical direction for success going forward.† †This foreword does not endorse any of the views expressed in the articles contained in this issue of the *American Journal of Tropical Medicine and Hygiene*.

